# Lipid Uptake by Alveolar Macrophages Drives Fibrotic Responses to Silica Dust

**DOI:** 10.1038/s41598-018-36875-2

**Published:** 2019-01-23

**Authors:** Xiaomin Hou, Ross Summer, Ziying Chen, Ying Tian, Jingjing Ma, Jie Cui, Xiaohui Hao, Lingli Guo, Hong Xu, Hongli Wang, Heliang Liu

**Affiliations:** 1 School of Public Health, North China University of Science and Technology, Tangshan, Hebei 063210 China; 2 Center for Translational Medicine and Jane and Leonard Korman Lung Center, Thomas Jefferson University, Philadelphia, PA 19107 USA; 3 Medical Research Center, Health, North China University of Science and Technology, Tangshan, Hebei 063210 China

## Abstract

Silicosis is a common occupational disease and represents a significant contributor to respiratory morbidity and mortality worldwide. Lipid-laden macrophages, or foam cells, are observed in the lungs of patients with silicosis but the mechanisms mediating their formation remain poorly understood. In this study, we sought to elucidate the mechanisms by which silica promotes foam cell formation in the lung, and to determine whether uptake of lipids alone is sufficient to drive TGF-β production by alveolar macrophages. Consistent with previous reports, we found that foam cells were markedly increased in the lungs of patients with silicosis and that these findings associated with both higher levels of intracellular lipid levels (oxidized LDL, ox-LDL) and elevated transcript levels for the lipid scavenger receptor CD36 and the nuclear receptor PPARγ. Employing a rat alveolar macrophage cell line, we found that exposure to silica dust or ox-LDL alone had a modest effect on the induction of foam cell formation and only silica was capable of inducing the production of TGF-β. In contrast, foam cell formation and TGF-β production were both dramatically increased when cells were exposed to a combination of silica dust and ox-LDL. Moreover, we found that these endpoints were markedly attenuated by either blocking CD36 or inhibiting the activity of PPARγ. Altogether, our findings suggest that foam cell formation and TGF-β production are driven by the simultaneous uptake of silica and lipids in alveolar macrophages and that strategies aimed at blocking lipid uptake by alveolar macrophages might be effective in ameliorating fibrotic responses to silica in the lung.

## Introduction

Silicosis is an occupational lung disease caused by exposure to crystalline silica dust (SiO_2_), which is a major constituent of soil, sand and most other types of rock. While silicosis is now a relatively uncommon respiratory condition in many regions of the world that have strict occupational safety standards, it remains a frequent cause of respiratory morbidity and mortality in many other regions of the world, including China. For example, in 2013 approximately 25,000 new cases of silicosis were diagnosed in China alone, which is a number that nearly equals the incidence of idiopathic pulmonary fibrosis (IPF) in the United States. However, unlike IPF, treatments for silicosis do not exist, illustrating the importance of gaining additional mechanistic insight into this condition.

Alveolar macrophages (AM) are the first line of defense against foreign substances entering the lower airways, and are essential for clearing silica dust from the lung^1^. Moreover, uptake of silica dust by AMs has been shown to play an important role in the pathobiology of silicosis, not only by driving the production of factors that contribute to lung inflammation but also by promoting the production of pro-fibrotic substances. For reasons that remain unclear, exposure to silica dust in both rodents and humans has been shown to induce the formation of foam cells, which are AMs that have accumulated increased amounts of intracellular lipids^2–4^. Although the role of foam cells in the pathobiology of silicosis remains unknown, recent reports have indicated that lipid uptake by AMs can by itself polarize cells to an M2 pro-reparative phenotype in the setting of bleomycin exposure, suggesting that foam cells may actually contribute to fibrotic remodeling in the silica-exposed lung.

To date, our understanding of the mechanisms contributing to foam cell formation are largely driven by work in the cardiovascular field^5–7^. In this context, it has been shown that the uptake of ox-LDL contributes significantly to the formation of foam cells and also triggers many of the events that underlie the development and progression of atherosclerosis^8,9^. Moreover, the uptake of ox-LDL has been shown to be mediated by several scavenger receptors on the surface of macrophages, most notably CD36, which is an 88-kDa glycoprotein responsible for an estimated 75% ox-LDL uptake^1,10^. Once taken up by macrophages, cholesterol and other lipids have been shown to activate fatty acid binding proteins and other intracellular lipid receptors, such as the liver x receptor (LXR) and PPARγ^11–14^. In turn, this activation drives transcriptional events that lead to the upregulation of various transporter proteins that then serve to facilitate the efflux of lipids from cells. Although the mechanisms mediating macrophage lipid clearance in the lung are less well-understood it has been shown that AMs express most, if not all, of the machinery involved in the uptake and clearance of lipids in atherosclerotic plaques. With this in mind, our primary objectives in this study were to determine whether silicosis alters the expression of the machinery involved in regulating lipid homeostasis in AMs, and to determine whether exposure to SiO_2_ or lipids alone can drive AMs to adopt a foam cell appearance and produce TGF-β.

## Results

### SiO_2_ induces foam cell formation in alveolar macrophage of the human lung

**Figure 1:**
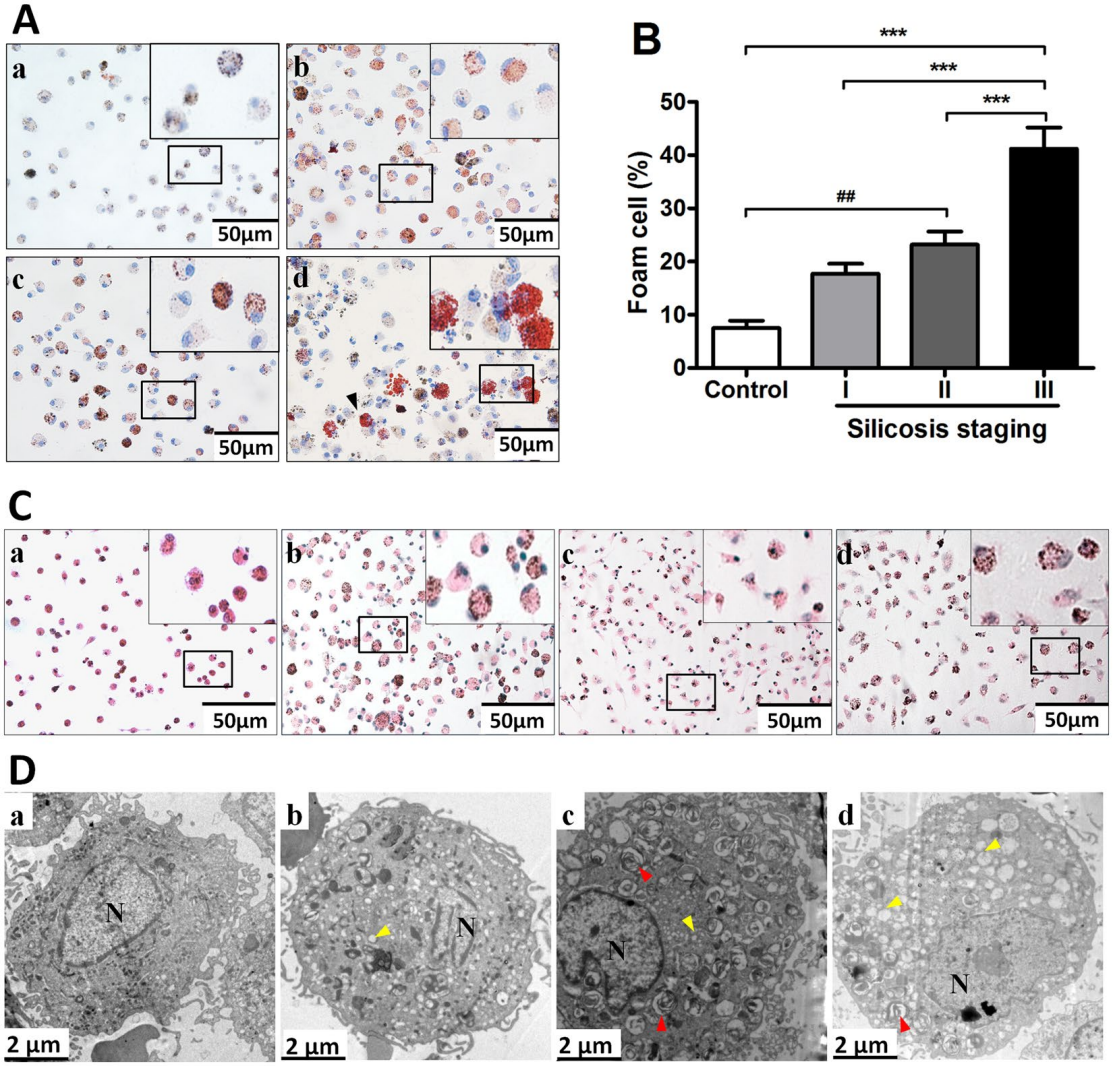
Alveolar macrophages (AM) from patients with and without silicosis are observed under the microscope. (**A**,**C**) AMs of (a) controls, (b) silicosis of stage I, (c) silicosis of stage II, (d) silicosis of stage III were viewed under inverted-microscope at magnification (400×) after Oil red O staining (black arrow) for neutral lipids or HE staining. (**B**) Percent positive foam cells were quantified. (**D**) AMs of (a) controls, (b) silicosis of stage I, (c) silicosis of stage II, (d) silicosis of stage III were observed by transmission electron microscopy from patients with and without silicosis. Red arrows highlight vacuolated areas consistent with lipid droplets.

The baseline characteristics of subjects in our study are shown in Table 1. We did not detect significant difference in age, smoking status or alcohol consumption between our groups (*P* < 0.05). As shown in Fig. 1, we found that AMs collected from patients with silicosis were on average larger, and exhibited a foamy appearance, as evidenced by the intense staining for neutral lipids (Oil Red O) in cells that were cytospun onto glass slides. Further, we found that the percentage of foam cells increased with the severity of disease and this was most apparent when cell morphology between individuals with stage III disease (*P* < 0.05) (Fig. 1A,B). Consistent with these findings, electron microscopy showed large number of low density vacuoles in the cytoplasm of AMs from patients with stage III disease, a findinglipids (Fig. 1D).

**Table 1:** Clinical characteristics of study subjects.

Clinical characteristics	Control (N = 8)	Silicosis I (N = 18)	Silicosis II (N = 17)	Silicosis III (N = 24)
Age	47.25 ± 5.8	46.33 ± 5.61	45.88 ± 5.44	48.92 ± 6.68
BMI	21.99 ± 3.45	23.51 ± 2.69	21.34 ± 2.55	22.86 ± 3.36
Dusting years		19.39 ± 10.47	12.18 ± 6.31	14.42 ± 7.25
Dust removal period		3.61 ± 6.03	6.0 ± 5.6	4.58 ± 5.51
Smokers/no smokers	6/2	17/1	14/3	16/8
Drinkers/no drinkers	5/3	13/5	15/2	15/9

Values are mean ± SD. *P*-values were calculated by one-way ANOVA tests (for age and BMI) or chi-square test (for smoker, drinker, out of the dust environment).

**Figure 2:**
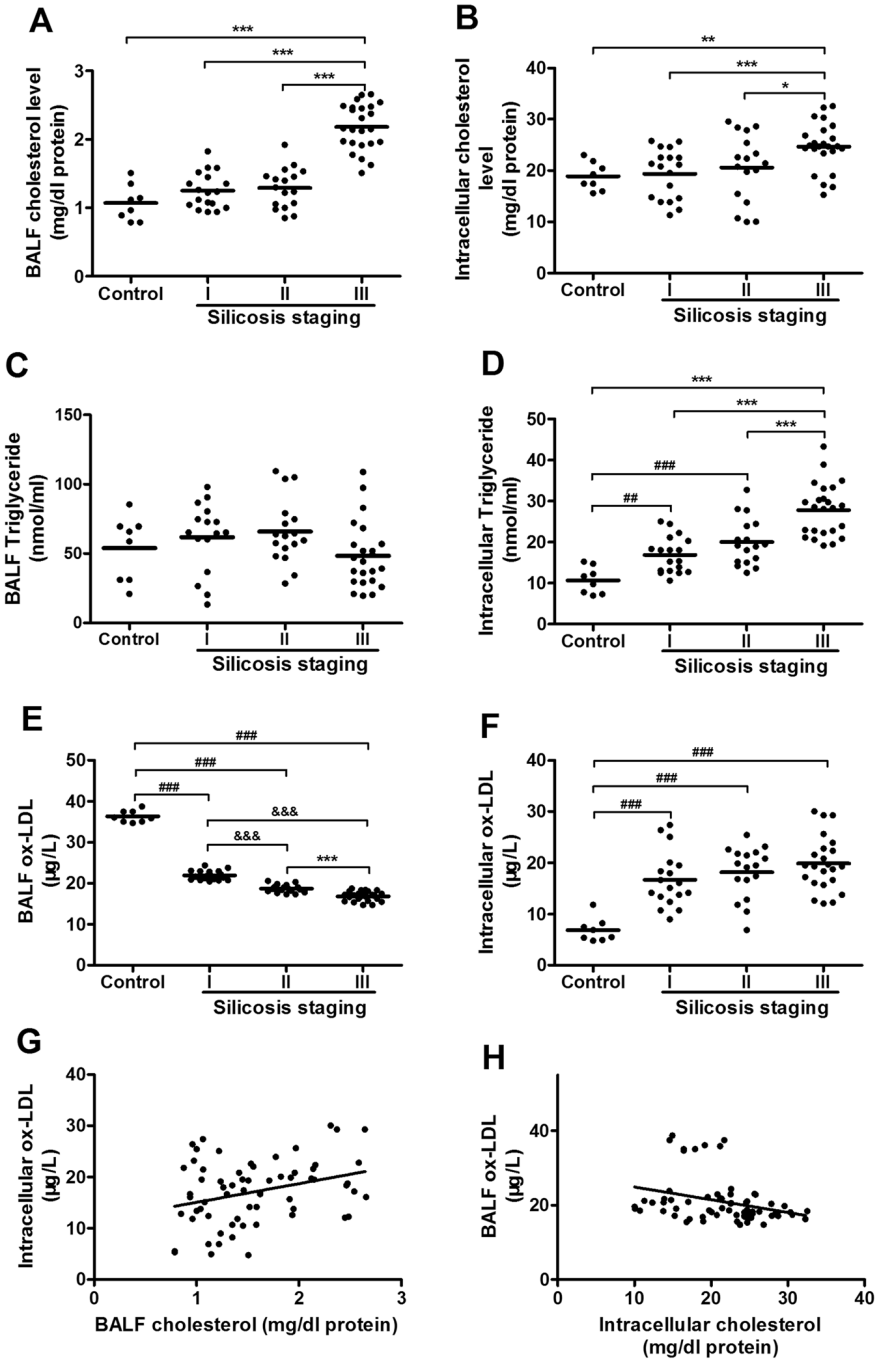
Lipid levels in bronchoalveolar lavage fluid and alveolar macrophages of subjects with and without silicosis. (**A**–**F**) Cholesterol, triglyceride and ox-LDL in BALF and AM in control subjects and patients with silicosis. (**G**,**H**) Dot plots showing correlation between ox-LDL levels and cholesterol levels in patients with silicosis. Data are presented as the mean ± SD from a duplicate of each sample and from three independent experiments (n = 3); ^#^*p* < 0.05; ^##^*p* < 0.01; ^###^*p* < 0.001 versus the control group, ^&^*p* < 0.05; ^&&^*p* < 0.01; ^&&&^*p* < 0.001 versus the silicosis I group, *p < 0.05; **p < 0.01; ***p < 0.001 versus silicosis III group.

**Figure 3:**
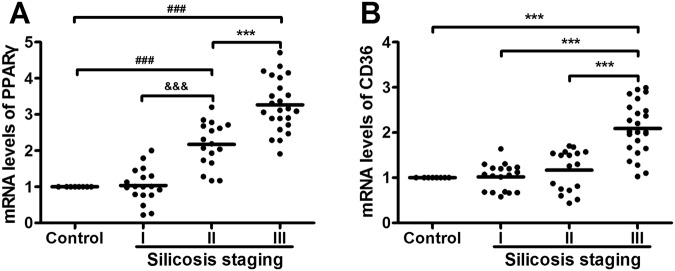
PPARγ and CD36 transcript levels in human AMs from individuals with and without silicosis. Values were mean ± SD of three independent experiments (n = 3). ^##^: *p* < 0.01; ^###^*p* < 0.001 versus the control group; ^&^*p* < 0.05; ^&&^*p* < 0.01; ^&&&^*p* < 0.001 versus the silicosis I group; *p < 0.05; **p < 0.01; ***p < 0.001 versus silicosis III group.

To delineate the type of lipids that accumulated we measured levels of cholesterol, triglycerides and ox-LDL in BAL fluid and AMs. As shown in Fig. 2, we found that cholesterol levels were increased in BALF and AMs silicosis, and that levels were associated with more severe disease. Indeed, cholesterol levels in stage III disease were markedly increased when compared to levels in all other stages (*P* < 0.05). Interestingly, triglyceride levels were not significantly increased in the BALF of patients with silicosis (*P* < 0.05), and levels in AMs showed only a modest elevation (*P* < 0.05). Like cholesterol, levels of ox-LDL were dramatically increased in cells collected from BAL fluid of silicosis patients (Fig. 2E), and levels were also increased at each stage of disease (*P* < 0.05). However, ox-LDL levels in the BAL fluid showed an inverse correlation with the severity of disease in contrast to cholesterol levels (*P* < 0.05). As shown in Fig. 2, we found a positive correlation (r = 0.343) between ox-LDL in AMs and cholesterol concentration in BALF (*P* < 0.05) (Fig. 2G) and a negative correlation (r = −0.335) between ox-LDL levels in the BALF and cholesterol levels in AMs (*P* < 0.05) (Fig. 2H). Consistent with cells that had accumulated intracellular lipids, we also found that transcript levels for the lipid scavenger receptor CD36 and the intracellular lipid receptor PPAR-γ were significantly increased in BAL cells from silicosis patients (Fig. 3A,B) (P < 0.05), supporting the notion that lipid homeostasis is altered in pulmonary silicosis.

### SiO_2_ augments foam cell formation in ox-LDL exposed NR8383 macrophages

**Figure 4:**
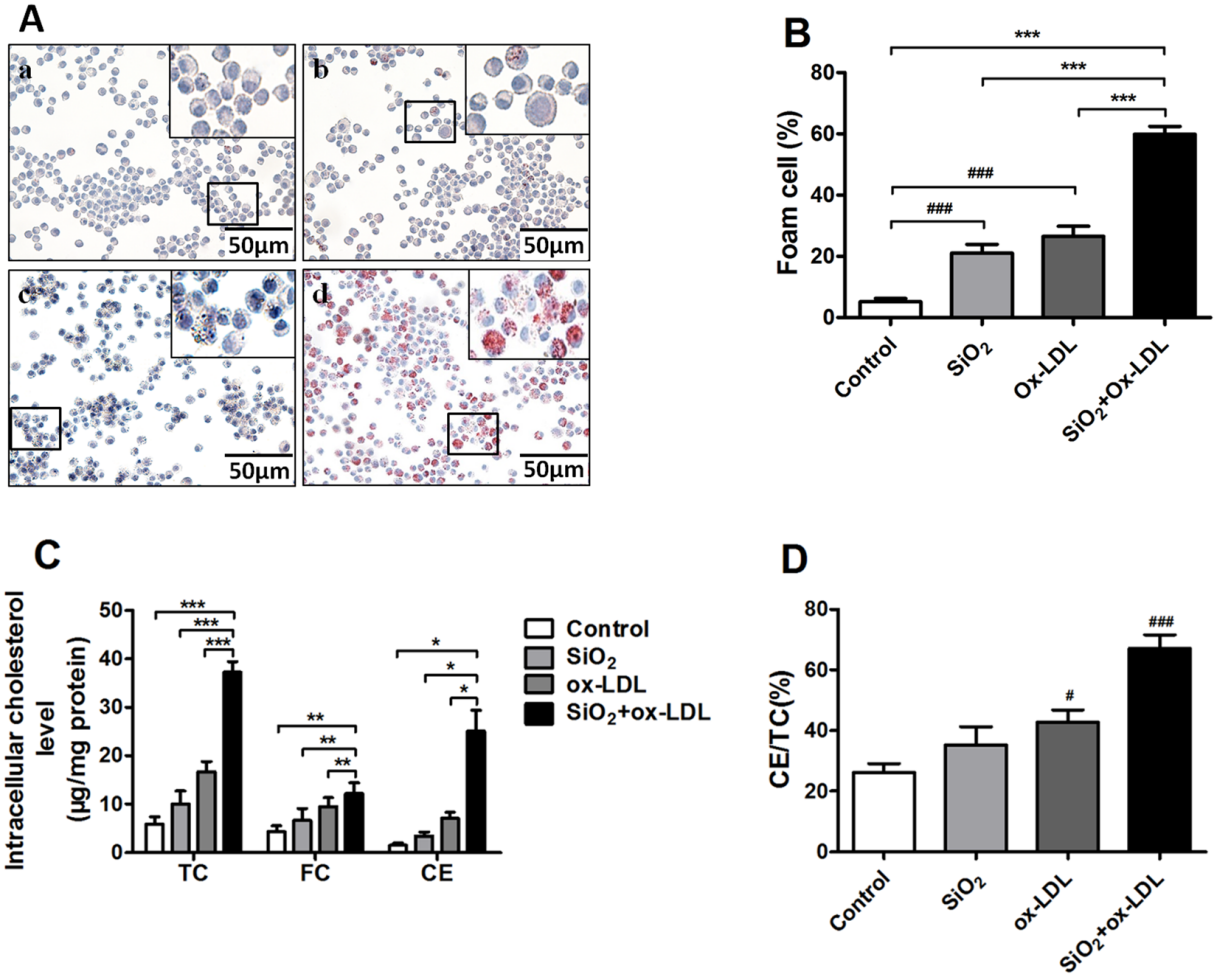
Combined exposure to SiO_2_ and ox-LDL induces NR8383 foam cell formation. (**A**) Foam cells readily formed in cells exposed to SiO_2_ plus ox-LDL (d) compared with control group (a), SiO_2_ group (b) and ox-LDL group (c) as evidenced by Oil Red O staining (400x). (**B**) Percent positive foam cells were quantified. (**C**) Total cholesterol (TC), free cholesterol (FC) and cholesterol ester (CE) levels in NR8383 cells in response to different conditions. (**C**) CE to TC ratios in NR8383 cells exposed to different conditions. Data are presented as the mean ± SD (n = 3). ^#^*p* < 0.05; ^###^*p* < 0.001 versus the control group; *p < 0.05; **p < 0.01; ***p < 0.001 versus the SiO_2_ + ox-LDL group.

To further study the effects of SiO_2_ on the formation of foam cells we employed an *in vitro* model system using NR8383 cells, a rat cell line often used to model human AMs in culture. Using these cells, we examined the impact of SiO_2,_ ox-LDL and combined exposures on the formation of foam cells. As shown in Fig. 4, we found that exposing cells to either low (50 μg/ml) or high (100 μg/ml) concentrations of SiO_2_ had only a modest effect on the formation of foam cells. Similarly, we found that exposing cells to lipids alone (ox-LDL) only modestly induced foam cell formation in NR8383 cells. In contrast, when cells were exposed to combined treatments of SiO_2_ and ox-LDL we detected a dramatic increase in the number and size of foam cells and this associated with a marked increase in levels of total cholesterol, free cholesterol and cholesterol esters in cells (Fig. 4C,D), suggesting that SiO_2_ augments the formation of foam cells in the confines of a lipid rich environment like the lung.

### SiO_2_ augments PPARγ and CD36 expression in ox-LDL exposed NR8383 cells

**Figure 5:**
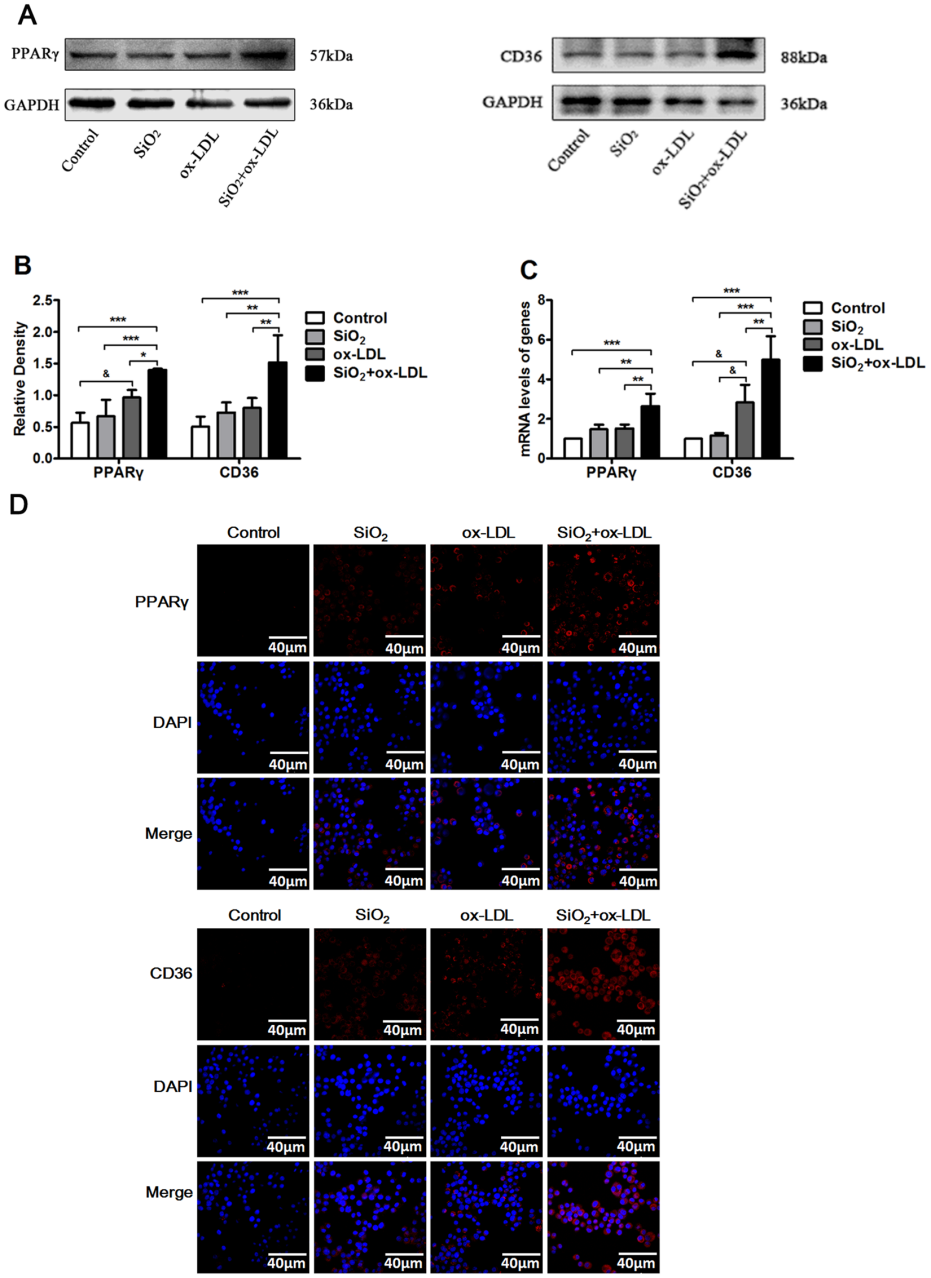
Combined treatment with SiO_2_ and ox-LDL induced CD36 and PPARγ expression in alveolar macrophages. (**A**) Western blot for CD36 and PPARγ in NR8383 cells exposed to different conditions. GAPDH was used as loading control. (**B**) Transcript levels for CD36 and PPARγ in NR8383 cells exposed to different conditions. (**C**,**D**) Immunofluorescence staining for CD36 and PPARγ in NR8383 cells exposed to different conditions. DAPI was used to stain nuclei (blue). All images show × 60 original magnifications. Data represent the mean ± SD from three independent experiments(n = 3). *p < 0.05; **p < 0.01; ***p < 0.001 versus the SiO_2_ + ox-LDL group. ^&^*p* < 0.05; ^&&^*p* < 0.01; ^&&&^*p* < 0.001 versus the ox-LDL group.

To investigate whether SiO_2_ can also alter expression of the machinery involved in regulating AM lipid levels we next examined transcript and protein levels for CD36 and PPARγ in NR8383 cells. Although SiO_2_ treatment alone did not significantly alter the expression of these genes, we found that transcript and protein levels of CD36 and PPARγ were both significantly increased in cells exposed to SiO_2_ plus ox-LDL (Fig. 5A–D). Together, these findings provide further support for the concept that SiO_2_ disrupts lipid homeostasis in AMs.

### SSO inhibits SiO_2_–induced foam cell formation in ox-LDL exposed NR8383 cell

**Figure 6:**
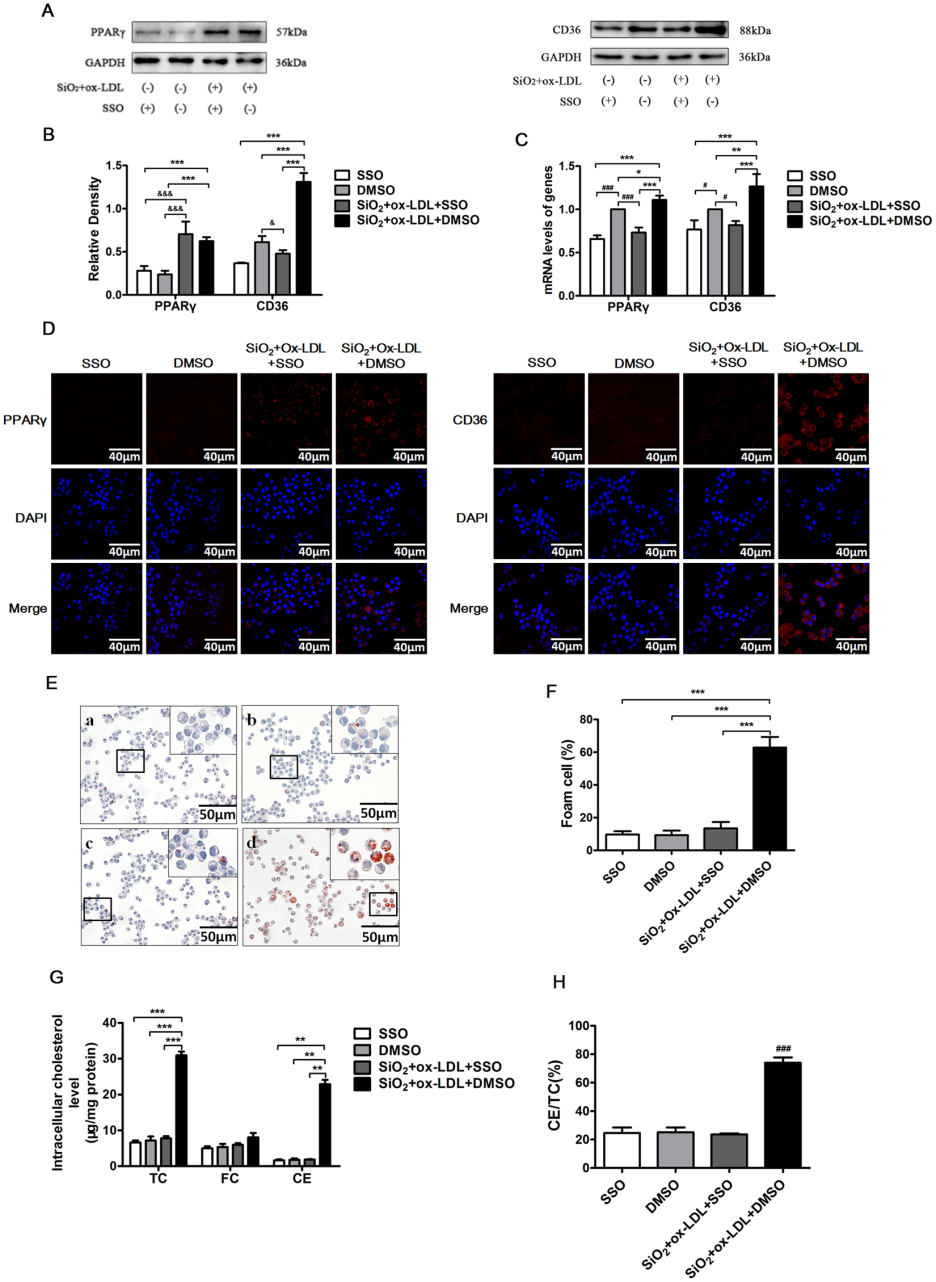
Inhibition of CD36 blocks foam cell formation in NR8383 cells. (**A**,**B**) NR8383 cells (1 × 10^6^/ml) were pretreated with CD36 inhibitor, SSO (10 μM) for 2 h and then treated with SiO_2_ + ox-LDL (50 μg/ml) isolated from different individuals for 36 h. Expression of CD36 and PPARγ transcript and protein levels were analyzed. (**C**,**D**) Immunofluorescent staining for CD36 and PPARγ (ret) and nuclei (blue) in NR8383 cells exposed to different conditions. (**E**) Oil Red O staining of NR8383 cells exposed to different conditions (400x). (**F**) Percent positive foam cells were quantified. (**G, H**) Intracellular total cholesterol (TC), free cholesterol (FC) and cholesterol ester (CE) levels or the ratio of CE to TC was determined in NR8383 cells exposed to different conditions. All data are expressed as mean ± SD from at least three independent experiments in triplicate. ^#^*p* < 0.05; ^###^*p* < 0.001 versus the DMSO group; ^&&&^*p* < 0.001 versus the SiO_2_ + ox-LDL + SSO group; *p < 0.05; **p < 0.01; ***p < 0.001 versus the SiO_2_ + ox-LDL + DMSO group.

To assess whether CD36 is required for the formation of foam cells in our *in vitro* model, we next treated NR8383 cells with a pharmacological inhibitor of CD36 called SSO. As shown in Fig. 6, we found that treatment with SSO effectively inhibited the upregulation of transcript and protein levels of CD36 (Fig. 6A–D) in the setting of SiO_2_ plus ox-LDL exposure. Further, we found that the downregulation of CD36 by SSO also associated with a marked reduction in intracellular lipid accumulation, as evidenced by the dramatic decrease in total cholesterol levels, CE/TC ratios (75.66% vs 21.42%) (Fig. 6H), and the number lipid droplets in SiO_2_ plus ox-LDL exposed NR8383 cells (*P* < 0.05) (Fig. 6E,F). Together, these findings suggest that blocking CD36 might be effective in inhibiting foam cell formation in the silica exposed lung.

### Blocking PPARγ activity reduces SiO_2_-induced foam cell formation

**Figure 7:**
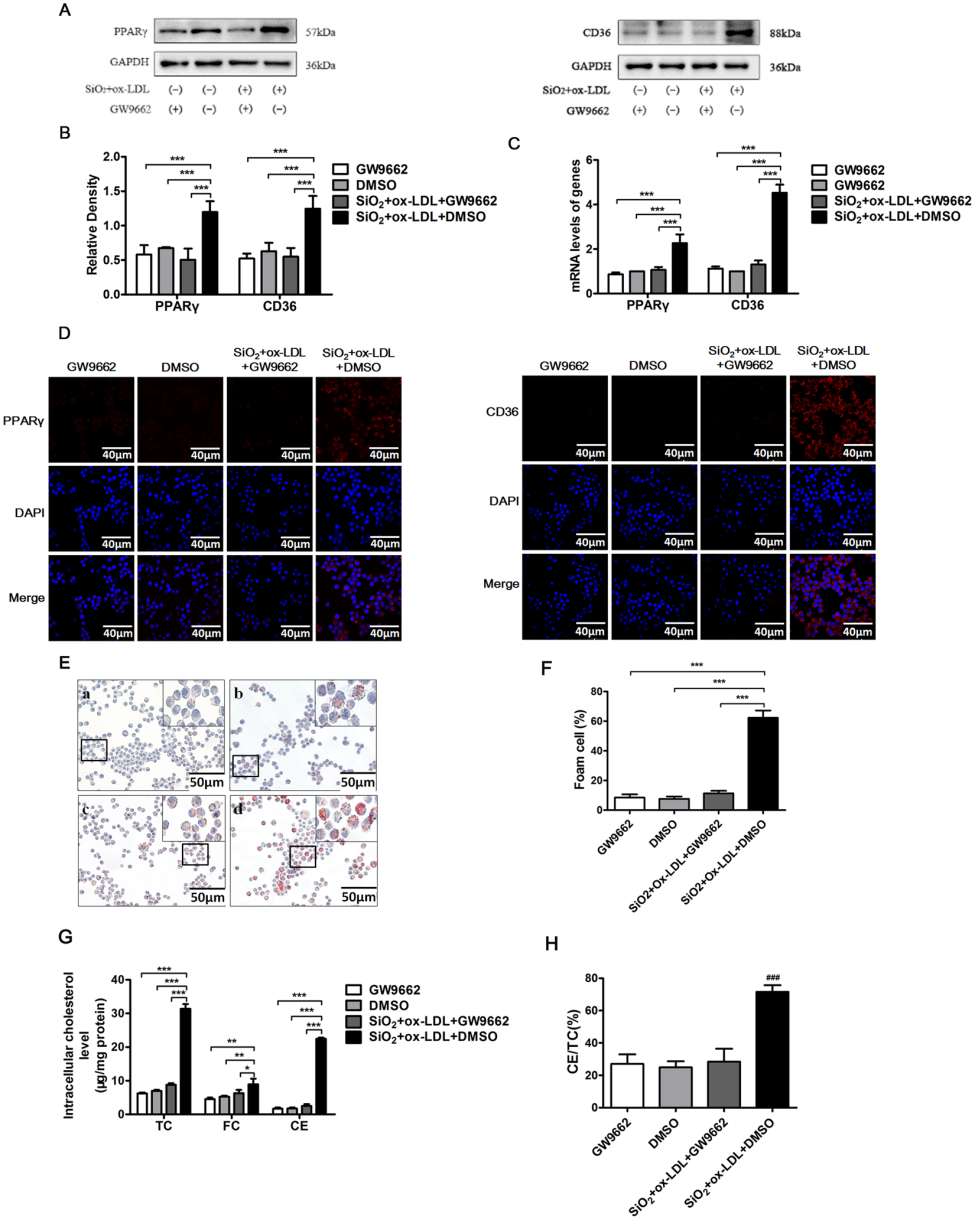
PPARγ inhibitors reduce foam cell formation in NR8383 cells. **(A–C)** Protein and transcript levels for CD36 and PPARγ in NR8383 cells exposed to different conditions. (**D)** Immunofluorescence images of CD36 and PPARγ (red) in NR8383 cells exposed to different conditions. DAPI was used to stain nuclei (blue, 600x). (**E)** Oil Red O staining in NR8383 cells exposed to different conditions (400x). (**F**) Percent positive foam cells were quantified. (**G**,**H**) Intracellular cholesterol levels and the ratio of CE to TC were determined in NR8383 cells exposed to different conditions. Data are expressed as mean ± SD from three independent experiments. ^###^*p* < 0.001 versus the control group; *p < 0.05; **p < 0.01; ***p < 0.001 versus the SiO_2_ + ox-LDL + DMSO group.

Next, to explore whether the PPARγ pathway is involved in regulating foam cell formation in AMs we pre-treated NR8383 cells with GW9662 for 2 h, which is a high-affinity PPARγ inhibitor known to abrogate PPARγ activation in various model systems^15^. As shown in Fig. 7, we found that treatment with GW9662 significantly reduced transcript and protein levels for PPARγ in NR8383 cells exposed to SiO_2_ plus ox-LDL (Fig. 7A–C). Interestingly, these findings also associated with a marked decrease in transcript and protein levels for CD36 (Fig. 7D). More importantly, we found that GW9662 dramatically reduced the quantity of intracellular lipids, as measured by the intensity of Oil Red O staining (Fig. 7E,F), levels of total cholesterol, free cholesterol and cholesterol esters in whole cell lysates and the ratio of cholesterol esters to total cholesterol in cultured cells (Fig. 7G,H). Together, these results show that the blocking PPARγ activity can be effective in inhibiting foam cell formation, perhaps by downregulating the expression of CD36 on AMs.

### Foam cell formation induces the production of TGF-β secretion

**Figure 8:**
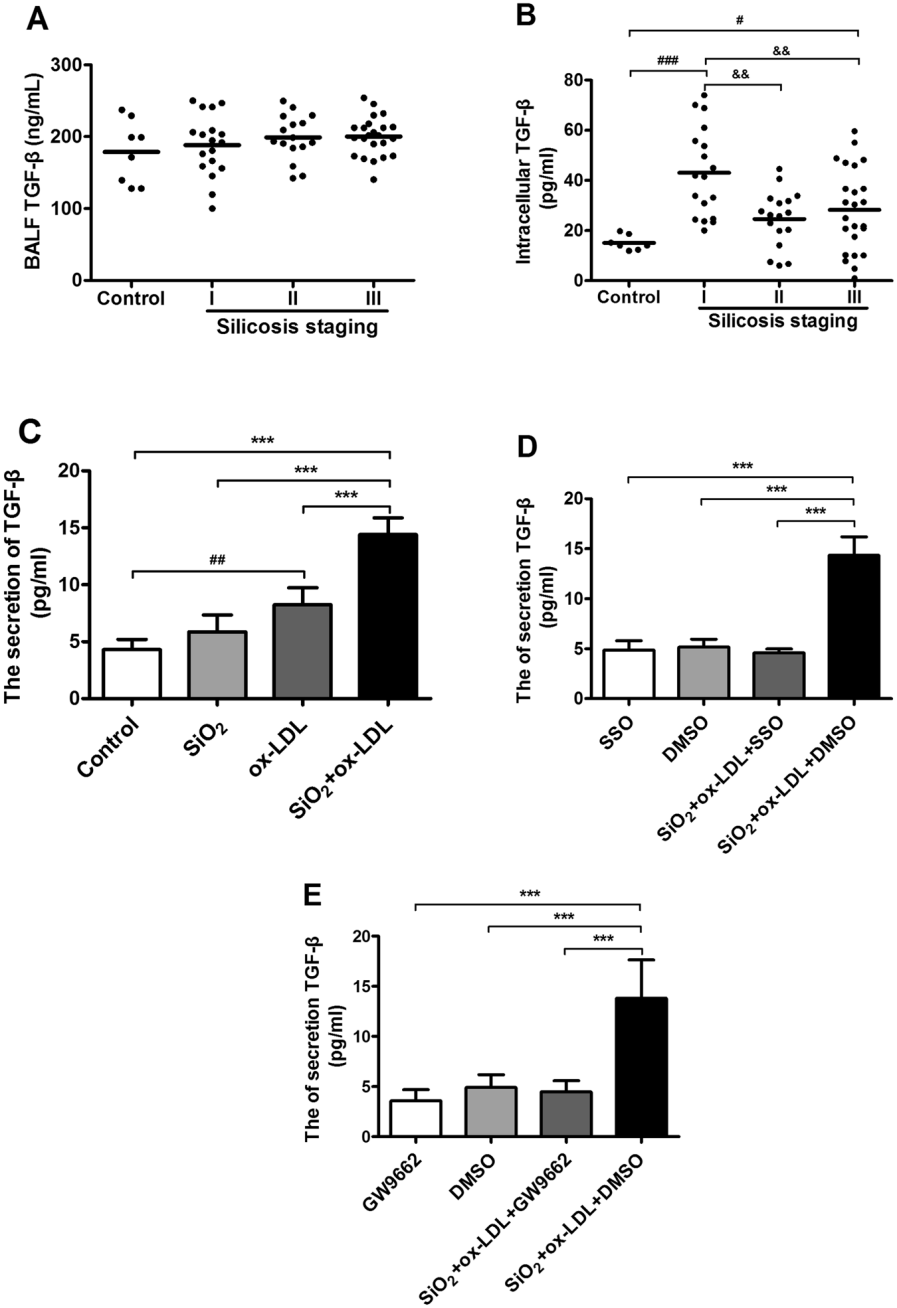
Foam cell formation induces the production of TGF-β. (**A**). TGF-β levels in BALF of patients with silicosis. (**B**) TGF-β levels in AMs of patients with silicosis. (**C**–**E**) The expression of TGF-βin NR8383 cell culture supernatants in different treatment groups. Data are expressed as mean ± SD from three independent experiments. ^#^*p* < 0.05; ^##^*p* < 0.01; ^###^*p* < 0.001 versus the control group; *p < 0.05; **p < 0.01; ***p < 0.001 versus the SiO_2_ + ox-LDL + DMSO group; ^&^*p* < 0.05; ^&&^*p* < 0.01; ^&&&^*p* < 0.001 versus the silicosis I group.

**Figure 9:**
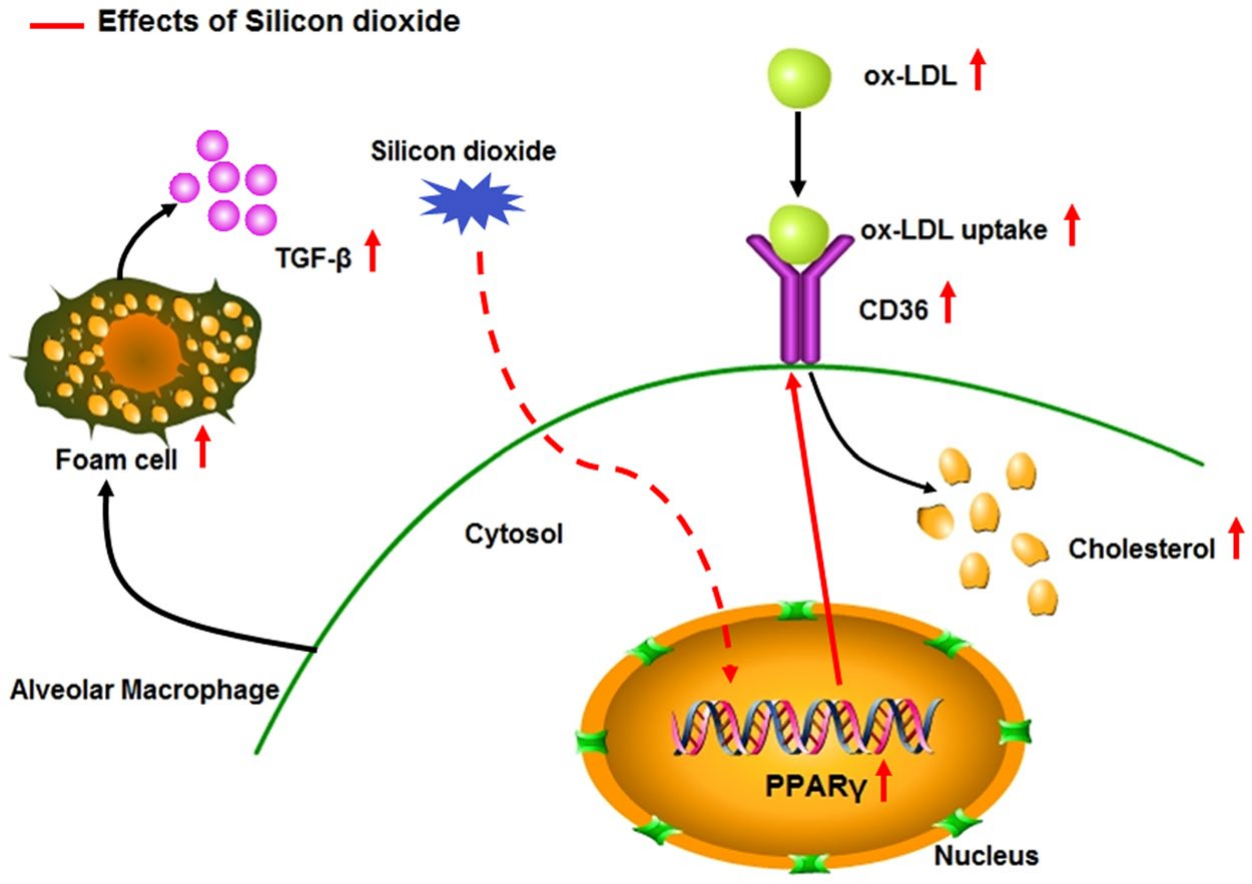
SiO_2_ promotes the uptake of ox-LDL from alveolar macrophages, perhaps augmenting CD36-PPARγ signaling.

Finally, to determine whether the formation of foam cells might actually contribute to fibrotic remodeling in the lung we compared levels of TGF-β in control and lipid-filled NR8383 cells. As shown in Fig. 8, we found that levels of TGF-β were significantly increased in BALF of patients with silicosis when compared to controls (*P* < 0.05). Similarly, we found that TGF-β production was also increased when NR8383 cells were exposed to combined treatments of SiO_2_ and ox-LDL but levels of TGF-β only increased marginally in cells exposed to ox-LDL alone. Importantly, we found that treatment of cells with either GW9662 or SSO to block PPARγ and CD36, respectively, effectively reduced the expression of TGF-β in SiO_2_ and ox-LDL exposed cells (*P* < 0.05), suggesting that pro-fibrotic responses by foam cells in the lungs of silicosis patients can be attenuated by these therapies (Fig. 9).

## Discussion

Foam cells are a hallmark feature of diseased tissues in a wide range of organs, including the lung, particularly in the setting of pulmonary fibrosis. In this study, we sought to elucidate the mechanisms by which foam cells form in the silica-exposed lung and to determine whether these cells might have a contributory role in the formation of pulmonary scar tissue. Interestingly, we found that SiO_2_ alone had only a marginal effect on the formation of foam cells but readily promoted their formation when placed in the context of a lipid-rich environment. Further, we found that combined exposures of SiO_2_ and ox-LDL not only markedly enhanced foam cell formation but also dramatically upregulated the production of TGF-β. Moreover, we showed that by blocking either the uptake of lipids or the activation of downstream signaling events in AMs we could effectively decrease TGF-β production in cultured cells^16–18^. Together, our findings suggest that targeting foam cell formation might be a novel strategy for treating silicosis and, perhaps, other fibrotic lung diseases as well.

While other groups have shown that foam cells accumulate in the lungs of patients with silicosis our work is the first to demonstrate that this is also associated with a dramatic increase in levels of ox-LDL within AMs. Further, our data indicate that uptake of ox-LDL synergizes with silica dust to drive fibrotic responses in AM. Of note, ox-LDL is a lipid found abundantly throughout the body but accumulates more in diseased tissues. When compared to non-oxidized LDL, ox-LDL tends to promote cellular dysfunction by activating inflammatory pathways, explaining why its accumulation has been linked to various diseases, including coronary artery disease and peripheral artery disease^19,20^. In these contexts, the uptake of ox-LDL drive cellular responses that not only lead to endothelial dysfunction but also to the progression of atherosclerosis^21–23^. Relevant to pulmonary fibrosis, it is also appreciated that a characteristic feature of foam cell-rich atherosclerotic plaques is a thick fibrous caps on the exterior surface^24^, suggesting that uptake of ox-LDL might also contribute to fibrotic remodeling in this disease.

Another important finding in our study was the observation that levels of CD36 are dramatically increased in AMs in response to SiO_2_ and ox-LDL exposure. CD36 belongs to the B family scavenger receptor^25^, a transmembrane protein receptor^26^ expressed on the surface of a variety of cells such as monocytes, macrophages, smooth muscle cells, and others. Because CD36 is a well-known lipid receptor, our findings suggest that the upregulation of CD36 on AMs may be an attempt to reduce extracellular lipid concentrations in the lung. Although this adaptive response may make sense in the setting of high levels of extracellular lipids, our findings also suggest that this response may contribute to fibrotic remodeling by augmenting TGF-β production in SiO_2_ and lipid exposed AMs. Notably, we are not the first group to demonstrate that levels of CD36 expression influence fibrotic responses. For example, Parks *et al*. showed that mice lacking CD36 were protected from developing pulmonary fibrosis to bleomycin^27^. Taken together, these findings suggest that pharmacological methods aimed at reducing lipid inflow to AMs may be effective in preventing or treating silicosis.

In addition to CD36, we found that PPARγ levels were also increased in AM exposed to SiO_2_ and ox-LDL^28–30^ and in human primary AMs in the late stages of silcosis. Why levels of PPARγ were increased only in the late stages of human disease is unclear but we speculate this can be explained by several factors. First, foam cells represent only a small minority of the total cell population of lung macrophages in patients with early stage silicosis (Fig. 1), making their overall contribution to PPARγ expression relatively small. Moreover, while all cells are exposed to silica in culture this is not the case when silica is inhaled into the airways of the lung. Finally, it is also possible that injured AMs are rapidly replaced by circulating monocytes in early stages of disease but trafficking in and out of the lung is compromised in later stages of pulmonary fibrosis, thereby leading to an accumulation of injured and foamy appearing cells in the lung.

While our study suggests that SiO_2_ promotes lipid uptake in the lipid-rich environment of the lung it is also important to note that intracellular lipid concentrations are also dependent on the efflux of lipids from the cytoplasm. This activity is dependent on one or more of the known ATP-dependent transporter proteins on the plasma membrane of AMs such as ABCG1 or ABCA1^31–34^. Because our study did not evaluate the impact of silica on the efflux of lipids, these types of studies will be important for future investigations.

Another notable finding in our study was the observation that TGF-β levels were only modestly increased in AMs when exposed to ox-LDL alone but levels markedly increased when cells were exposed to combined treatments of SiO_2_ plus ox-LDL. These findings indicate that SiO_2_ somehow synergizes with ox-LDL to drive fibrotic responses. Importantly, we showed that blocking lipid uptake/signaling by either inhibiting CD36 or PPARγ was highly effective in reducing TGF-β to these combined exposures, suggesting targeting these molecules might be effective in the treatment of silicosis. That said, we recognize the best way to reduce the burden of silicosis would be to prevent disease by limiting exposure to silica dust. However, effective preventive strategies have yet to be employed in many regions of the world, making it important to consider novel treatment approaches for these individuals.

We would like to point out that our study has several notable limitations. First, we recognize that our study was performed only on individuals from China, making it difficult to interpret whether our findings our applicable to more diverse populations. Also, our *in vitro* studies were performed using a rat macrophage cell line rather than primary cells. Thus, at some point, our findings will ultimately need to be confirmed using primary macrophages from the human lung.

In conclusion, our work describes a novel mechanism by which SiO_2_ might conspire with lipids to drive foam cell formation and trigger the production of pro-fibrotic substances and that targeting foam cells might be a new avenue for the prevention or treatment of silicosis. We believe these findings have broad implications for the field of pulmonary medicine and other fields in which the formation of foam cells is a characteristic pathological feature.

## Methods

### Study subjects

Study subjects were seen in the Beidaihe Chinese coal workers nursing home. All patients were referred to our hospital for a one-time whole lung lavage. Silicosis was diagnosed based on GBZ-70–2009 “Diagnostic criteria of pneumoconiosis”. Patients were excluded from the study for any of the following: (1) active pulmonary tuberculosis; (2) known heart disease; (3) bacterial pneumonia; (4) liver cirrhosis or hyperlipidemia. By nature of fact that silicosis is mostly a disease of men, all subjects in our study were males. The control group of 8 cases included 5 normal subjects and 3 cases of aspiration pneumonitis. Due to the challenges of recruiting normal subjects for lung lavage we were only able to obtain samples from 8 controls. This study was approved by the Clinical Trial and Ethics Committee of North China University of Science and Technology, and all participants provided written informed consent.

### Chemicals and reagents

Ham’s F12K, Roswell Park Memorial Institute-1640 (RPMI-1640), fetal bovine serum (FBS) and penicillin-streptomycin were purchased from Invitrogen (Gibco, USA). SiO_2_ (particle size: 0.5 ∼ 10.0 μm, 80% of particle size is 1.0 ∼ 5.0 μm), GW9662, Dimethyl sulfoxide (DMSO), and Oil red O were from Sigma. Ox-LDL was purchased from Yiyuan Biotechnologies (Guangzhou, China). SSO was from Toronto. Primary antibody of CD36 (1:8000 dilution), PPARγ (1:800 dilution), GAPDH (1:2500 dilution) and conjugated with horseradish peroxidase (1:1000 dilution) were purchased from Abcam (Abcam, Cambridge, MA, USA). Cell Viability Assay Kit MTS was from Promage in the United States. Cholesterol, triglyceride and ox-LDL ELISA kits are available at Biovision in the United States. TGF-β were purchased from ABclone (USA). Total Cholesterol and Free Cholesterol Kit were obtained at Beijing Puli Lai Gene Technology Co, Ltd. Reverse transcription kit and SYBR Green qPCR reaction kits were from Takara, Japan.

### Whole lung lavage and isolation of bronchoalveolar lavage fluid (BALF) and AM

Whole lung lavage was performed per published protocols. In China, whole lung lavage is used to treat silicosis. In brief, subjects are placed under general anesthesia and intubated with a large-capacity double-lumen tube. Unilateral lung lavage is then performed using 10000 ml–12000 ml in 500 ml aliquots. Recovered fluid is sterile- filtered and aliquots are removed for conventional cell counting and staining as well as isolation of cell pellets and BAL fluid for later analysis.

### Cell culture

The NR8383 rat alveolar macrophage cell line was purchased from Chinese Academy of Sciences Shanghai Cell Bank. The cell density was adjusted to 2 × 10^5^ cells /ml with F12K medium (adding 1% penicillin and streptomycin) with volume fraction of 15.0% fetal bovine serum, and cultured in incubator. The medium was changed every 2 to 3 days.

### Histology

Hemotoxylin and eosin staining and Oil red O staining were performed as previously described.

### Electron microscopy

Human alveolar macrophage pellets were fixed using standard protocols and as previously described.

### Enzyme-Linked Immunosorbent Assay (ELISA)

Cholesterol, triglyceride, ox-LDL and TGF-β levels were measured in AMs and BALF using commercially available ELISA kits. Experimental steps were performed according to manufacturer’s protocol.

### Measurement of total cholesterol, free cholesterol and cholesterol ester in NR8383

The total cholesterol (TC), free cholesterol (FC) levels were determined according to the Kit instructions at 450 nm by a microplate reader, and the proportion of intracellular cholesterol ester (CE) was calculated. [CE% = (TC − FC)/TC].

### Western blot analysis

Total protein concentration was determined by a BCA assay kit. The samples (30 μg) were then heated denatured and separated on 10% SDS-PAGE before being transferred to a PVDF membrane. Membrane was blocked with 3.5% skim milk containing 0.05% Tween-20 (TBST) for 3 hour, followed by incubation with primary antibodies at 4 °C overnight. The incubation time for secondary antibody was 2 hour at room temperature. An enhanced Chemiluminescence detection system was used for detection.

### Real-time quantitative real-time PCR

Total cellular RNA was isolated from treated cells using Trizol. RNA concentration and purity were determined by absorbance measurements at 260 nm and 280 nm (A260/A280 = 1.8–2.1) using a NanoDrop 8000 Spectrophotometer. cDNA was synthesized using the PrimeScript^TM^ RT reagent Kit. The transcribed cDNA was amplified with 200 ng cDNA using SYBR Green detection chemistry on the ABI 7500 Sequence Detection System. Relative expression levels were determined using the 2^−ΔΔCt^ method. The sequences of PCR primers used in this study are as follow (Table 2).

**Table 2:** Sequences of primers and size of products for PCR.

Gene	Sequences of primers	Amplicon
PPARγ	Forward 5′-CACGGTTGATTTCTCCAGCATTTC-3′	135
	Reverse 5′- GCAGGCTCTACTTTGATCGCACT-3′	
CD36	Forward 5′-TTCTCATGCCGGTTGGAGACCTA-3′	172
	Reverse 5′- TTGCTGCTATTCTTTGCCACTTC-3′	
GAPDH	Forward 5′-CCATCACGCCACAGTTTCC-3′	357
	Reverse 5′-ATCAGCAATGCCTCCTGCA-3′	

### Immunofluorescence for PPARγ and CD36 expression

Cells were fixed with 4% (w/v) paraformaldehyde for 15 min at room temperature, blocked with BSA, followed by blocking with normal goat serum for 60 min, and then incubated with antibody (1:100) overnight at 4 °C. After incubation with a secondary TRITC conjugated antibody and DAPI, the cells were washed with PBS, mounted in anti-fade reagent and then observed using laser scanning confocal microscope.

### Statistical analysis

Statistical analysis was performed using SPSS statistical software, version 22.0. All the experiments were repeated at least three times. Data were expressed as means ± standard deviations (SD). Inter-group differences were analyzed using one-way ANOVA for the comparison of three or more groups. The value of *p* < 0.05 was considered statistically significant.
